# Curcumin in Stevens-Johnsons Syndrome: Culprit or Bystander?

**DOI:** 10.1097/WOX.0b013e31819f20d4

**Published:** 2009-04-15

**Authors:** Carla Irani, Fadi Haddad, Georges Maalouly, Rita Nemnoum

## *To the Editor*

Stevens-Johnson syndrome (SJS) represents an immunologically mediated disease due to hypersensitivity to drugs or infections. It is a diffuse, severe mucocutaneous eruption involving mucosal surfaces, with or without visceral involvement or fever. Its pathogenesis is not well understood but mechanisms such as keratinocyte apoptosis are well documented. We report the case of a woman with SJS after repeated ingestion of curcumin. A 50-year-old woman was admitted to the hospital for a generalized pruritic rash that started a week before her admission. She had no history of allergy, drug consumption, fever, or upper respiratory infection. She recently started adding 2 teaspoons (5 g) of curcumin to her meals daily after reading about its antioxidant properties.

**Figure 1:**
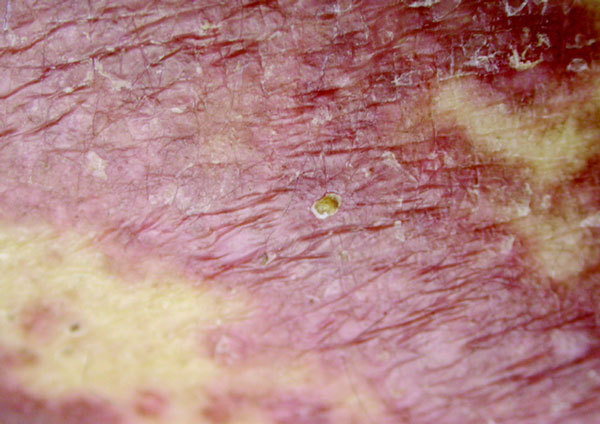
**Widespread maculopapular skin rash**.

**Figure 2:**
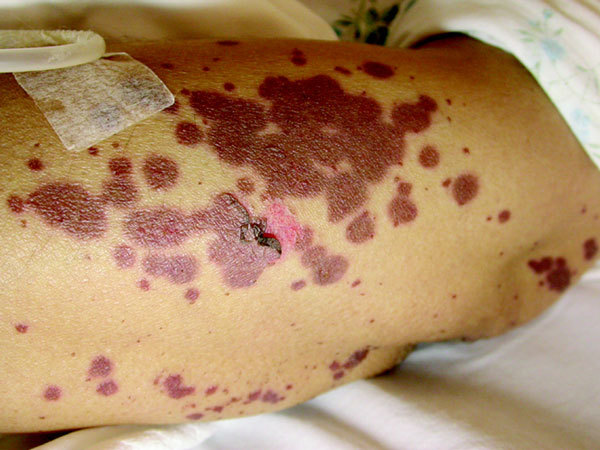
**Nikolsky's sign**.

On admission, vital signs were normal, she was afebrile, and she had a diffuse, maculopapular rash with some areas of desquamation. Erosions were present in the oral cavity. She had severe conjunctivitis and a positive Nikolsky's sign (Figures 1, 2).

**Figure 3:**
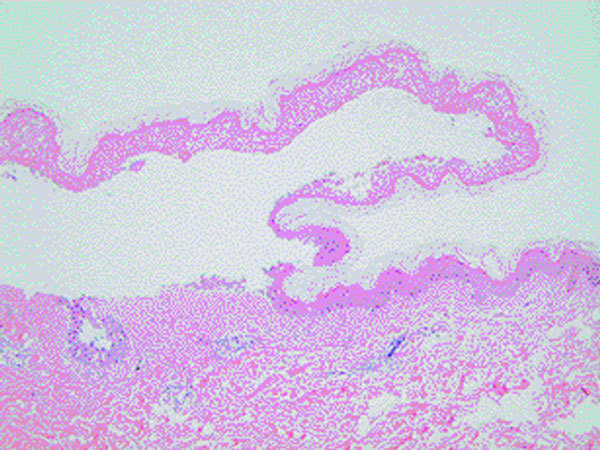
**Skin biopsy with epidermal detachment and lymphocyte infiltration in the dermis**.

Laboratory studies showed normal hemoglobin, white blood cell count, and platelets. Biochemistry evaluation, C-reactive protein, and chest x-ray were normal. Serum protein electrophoresis showed hypergammaglobulinemia. Serology of cytomegalovirus and Herpes simplex virus showed high specific IgG and low IgM. IgM antimycoplasma pneumonia was low threshold with a negative IgG. Skin biopsy confirmed the presence of SJS (Figure 3). The patient was treated with antihistamines and intravenous methylprednisolone starting at 4 mg/kg per day for 5 days and then tapered very slowly as the eruption resolved. She was discharged after clinical remission on oral prednisone tapered and antihistamines.

In SJS, there is epidermal detachment less than 30% of the total body surface, and an increase of keratinocyte apoptosis, which is rare in the normal epidermis. Keratinocytes normally express the death receptor Fas (CD95), which will be activated after upregulation of keratinocyte FasL expression and in the presence of a trigger. Numerous mediators of keratinocyte apoptosis have been suggested [1]. The main causes of SJS are drugs and less frequently viral infections. Other possible triggers are systemic diseases, herbal medicines, and food. In a review of mycoplasma infections associated with SJS,[2] most of the 70 patients were children and young adults with a predominance of the male sex. Most patients had prodromal symptoms of an upper respiratory tract infection and underlying pneumonia. Our patient was asymptomatic in that regard. Because the borderline positive IgM antimycoplasma is not confirmatory of the infection, hypergammaglobulinemia, seen in acute inflammatory states such as SJS, is a possible explanation of positive mycoplasma, herpes virus, and cytomegalovirus serology. A polymerase chain reaction to mycoplasma, not done in our patient, would have been most appropriate to make the diagnosis.

Turmeric is a bright yellow spice from the root of *Curcuma longa*, used for centuries in India as a spice, food preservative, or herbal medicine. Turmeric extracts are known to be safe in animal studies, although hepatotoxicity has been reported after ingesting large amounts [3]. Curcumin has a potential therapeutic value for controlling allergic responses resulting from exposure to allergens by diminishing the Th2 profile,[4] and it induces keratinocytes apoptosis through different pathways including the Fas receptor and NF-*κ*B [5]. Recently, Lee et al [6] showed that curcumin inhibits syk kinase-dependent signaling events in mast cells and contribute to its antiallergic activity. However, the pro-apoptotic and antiproliferative properties of curcumin may hypothetically facilitate or even cause the onset of SJS, an allergic reaction independent of mast cells. Whether the curcumin was the culprit of SJS in our case or a bystander of an unconfirmed infection, the apoptotic hypothesis seems a possible mechanism linking curcumin to SJS. More investigations are needed to define curcumin as an immunomodulator, a potential allergen, or both.

Carla Irani, MD

Fadi Haddad, MD

Georges Maalouly, MD

Rita Nemnoum, MD
